# Continuous Reusability using Immobilized HasApf in Chemoenzymatic Deracemization: A New Heterogeneous Enzyme Catalysis

**DOI:** 10.3390/biom6040041

**Published:** 2016-10-25

**Authors:** Hiroyuki Nagaoka

**Affiliations:** Sanyo Shokuhin Co., Ltd. R & D, 555-4 Asakura, Maebashi, Gunma 371-0811, Japan; hnagaoka@sanyofoods.co.jp; Tel.: +81-27-220-3471

**Keywords:** immobilized HasA, iron electron-transfer system, porous ceramic particle, continuous reusability, chemoenzymatic deracemization

## Abstract

This study found that the calibration curve of heme acquisition system A (HasA, a new reactive active species) immobilized by a porous ceramic particle (ImHApf; immobilized HasA from *Pseudomonas fluorescens*) can be constructed in the range of 1750–1450 cm^−1^ using Fourier transform infrared spectroscopy (FTIR) analysis, and evaluated its catalytic efficiency. In the asymmetric oxidation of *rac*-1-(6-methoxynaphthalen-2-yl)ethanol (*rac*-**1**: a naproxen precursor), a product ketone from the (*R*)-isomer is desymmetrized using NaBH_4_ and continuously reused even if treated with an organic solvent in 50 mM glycine–NaOH buffer at 40 °C in the absence of nicotinamide adenine dinucleotide (NAD(P)), leading to >99% enantiomeric excess and >90% chemical yield; the activity was calculated at 0.74 ± 0.03 mU/(mg·min) and the turnover number was determined to be approximately 2 × 10^6^. It was confirmed that the other *sec*-alcohols such as *rac*-1-(2-naphthyl)ethanol (*rac*-**2**) and *m*- and *p*-substituted *rac*-1-phenyl ethanols (*rac*-**3ab**–**6ab**) using ImHApf can also yield a single stereoisomer from a racemate. Therefore, HasA immobilization can be expected to become an important tool for building an environmentally friendly system that promotes industrial sustainability.

## 1. Introduction

The asymmetric oxidation of secondary alcohols (*sec-*alcohols) using a heme-binding protein incorporating an iron electron-transfer system as an enzymatic catalyst has been established in organic synthesis [1], whereas the mechanism of heme uptake by heme acquisition system A (HasA), a heme-binding protein which is secreted by various gram-negative pathogens, has been reported [2,3,4]. The advantages of immobilized or heterogeneous biocatalysts over those that are “free” in solution are well known and include stability, reusability, convenience in continuous operation, and volumetric productivity [5]; there is increasing interest in the use of tougher polymeric materials, particularly inorganic ceramic supports. For example, inorganic carriers for enzyme support represent an important topic in organic synthesis, and porous ceramic particles prepared from kaolin minerals are well suited for the enzymatic production of various biochemicals, such as pharmaceuticals, agricultural chemicals, and their precursors [6]. If the asymmetric oxidation system of HasA could be immobilized by a porous ceramic particle (ImHApf; immobilized HasA from *Pseudomonas fluorescens*), this new system would not only be able to overcome comparison to dehydrogenase enzymatic systems using coenzyme nicotinamide adenine dinucleotide (NAD(P)), but could also be used to evaluate the efficiency and turnover of HasA in ImHApf using Fourier transform infrared spectroscopy (FTIR) analysis [7].

**Scheme 1:**
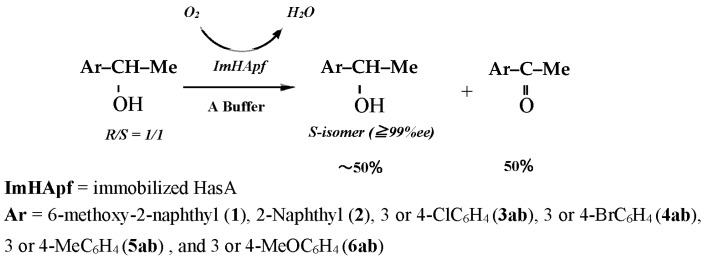
Kinetic resolution of *rac*-**1** or *rac*-**2** or *rac*-**3ab**–**6ab** using immobilized HasA from *Pseudomonas fluorescens* (ImHApf). ee: enantiomeric excess.

Over the past decade, the use of biomaterials for asymmetric oxidation has been examined [8], i.e., the redox activity of the pea protein immobilized by Ca^2+^-alginate gel (IPP; immobilized pea protein) is available for the kinetic resolution of *sec-*alcohols [9], as shown in Scheme 1, e.g., *rac*-1-(6-methoxynaphthalen-2-yl)ethanol (*rac*-**1**: a naproxen precursor), *rac*-1-(2-naphthyl)ethanol (*rac*-**2**), *rac*-1-octen-3-ol, and *m*- and *p*-substituted *rac*-1-phenyl ethanols (*rac*-**3ab**–**6ab**) [10]. Specifically, membrane-bound enzymes (MEs) eluted from IPP after aeration can be activated by a glycine–NaOH buffer (pH 9.0–10.0) in the absence of NAD(P) [11], and they can be applied to turnover kinetic resolutions, synthesizing (*S*)-(+)-**1** via a polyethylene glycol-coated ME [12]. The key reaction for asymmetric oxidation lies in an iron electron transfer system based on an oxygen-driven catalytic system [13], and the nature of the species showed 93% similarity with a 20.853 kDa hemophore HasA expressed by *Escherichia coli* BL21(DE3) (HasApf) after using an N-terminal sequence comparison [14]. Therefore, it is suggested that the successive asymmetric catalytic events can be regenerated using an incorporated iron electron transfer system [15], which is similar to that utilized by the oxygen-driven cytochrome P450 [16]. There are some structural differences between cytochrome P450 and HasA: namely, the key structural features of cytochrome P450 are that heme iron is coordinated by a cysteine ligand in the proximal site [17] and is open for oxygen binding in the distal site [18], resulting in a better system to activate oxygen and catalyze oxidations, whereas the set of HasA ligands appears to create an unlikely system because the heme iron is already coordinated [19], that is, a histidine is the ligand in the proximal site, and a tyrosine is coordinated by the distal site [20]. Therefore, it has been previously demonstrated that HasA functions at the distal site, so that oxygen will bind for oxygen activation to be oxoferric (Fe(III)–O–O^−^) species and so that it will coordinate solely in the promotion of oxoferryl (Fe(IV)=O) species. Moreover, one study showed that the further explorations of HasA should be regarding efficiency, turnover number, or enzyme loading in comparison with other known systems for this transformation or similar transformations [21].

The construction of deracemization methods that obtain a single enantiomer from a *sec-*alcohol [22] is classified according to the stereochemical course of enzymatic and chemical reactions [23], and the one-pot deracemization of biochemicals, such as pharmaceuticals, agricultural chemicals, and their precursors, is emphasized [24]. Two notable issues are whether ImHApf can be evaluated using a FTIR calibration curve and whether cyclic deracemization with continuous reusability can occur via ImHApf [25]. Therefore, this study aimed to (1) construct a FTIR calibration curve using different concentrations of HasA in ImHApf; (2) evaluate the turnover number and activity; (3) clarify the cyclic deracemization process using ImHApf and NaBH_4_; and (4) present ImHApf as a new asymmetric oxidation catalysis tool.

## 2. Results

### 2.1. Application to the Immobilization of HasApf

#### 2.1.1. Immobilization of HasApf with a Toyonite

**Figure 1:**
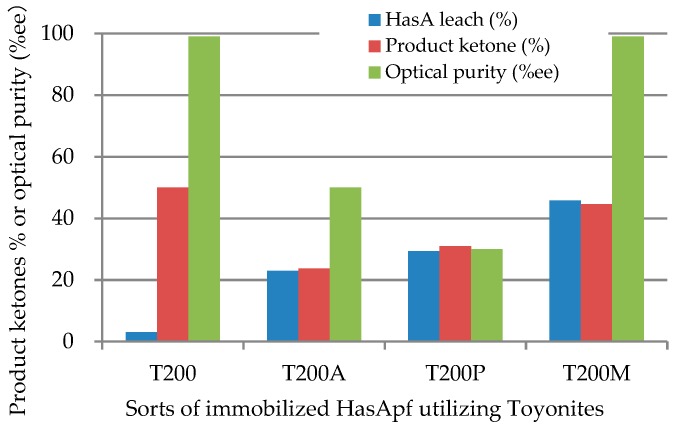
Asymmetric oxidation activity using immobilized heme acquisition system A expressed by *Escherichia coli* BL21(DE3) (ImHApf) and *rac*-**1** (0.8 mM) in a 50 mM glycine–NaOH buffer (4.0 mL); HasA leach (blue: 100% maximum) and product ketone (red: 50% maximum) were monitored, suggesting that the degree of heme acquisition system A (HasA) leaching from ImHApf may be in the lowest level of Toyonites (i.e., T200 < T200A < T200P < T200M). ee: enantiomeric excess.

The Toyonite family, consisting of porous ceramic particles prepared from kaolin minerals, exhibits differences in adsorption onto hydrophobic or hydrophilic surfaces because of the modification of four types of organic functional groups. As shown in Figure 1, the Toyonites (1.0 g) were suspended in a HasApf solution (18 mg/10 mL) containing a 50 mM potassium phosphate buffer (pH 9.0) for 50 h, and ImHApf was synthesized in the four Toyonites. The four Toyonites (2 mg each) were reacted with *rac*-**1** (0.8 mM:0.4 mg) in a 50 mM glycine–NaOH buffer (4.0 mL) for 50 h. The results indicate that Toyonite 200 was most suitable for HasA immobilization/adsorption because of a resulting lower HasA leach (referring to the degree of HasA (%) that remains in the filtrate after filtering the suspension) and a higher product ketone and optical purity; thus, HasA can be effectively immobilized/adsorbed onto Toyonite, which features an organic hydroxyl group (in the case of Toyonite 200), a methacryloyloxy (Toyonite 200M), phenylamino (Toyonite 200P), or amino group (Toyonite 200A) [5]. This result means that HasA may be a hydrophilic moiety in the structure and that Toyonite 200 plays an important role in the immobilization. In addition, it was speculated that, compared with other immobilization techniques (e.g., utilizing a 1.0% (v/v) aqueous CaCl_2_ solution for Ca^2+^-alginate gel beads [9] or a 0.5% (v/v) glutaraldehyde solution for cross-linking [10]), HasA can be simply and effectively immobilized into porous ceramic particles (particularly, Toyonite 200) without additional chemicals.

#### 2.1.2. FTIR Analysis for the Detection of HasA Spectra in ImHApf

**Figure 2:**
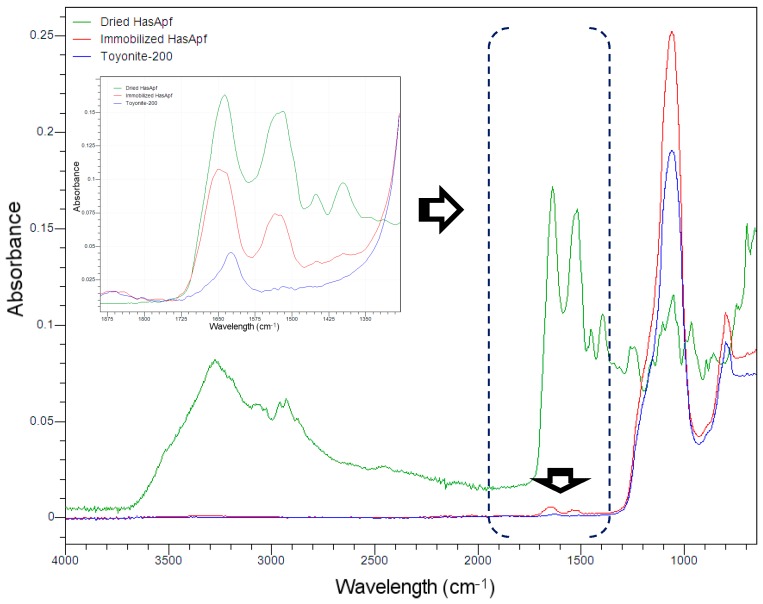
Different Fourier transform infrared (FTIR) spectra of dried heme acquisition system A expressed by *Escherichia coli* BL21(DE3) (HasApf), immobilized HasApf, or Toyonite 200. HasA spectra in ImHApf were located in the range of 1750–1450 cm^−1^.

For the first time, a calibration curve of HasA was constructed using FTIR, a portable attenuated total reflection (ATR) instrument (A2 Technologies, ML version: S.T. Japan Inc., Tokyo, Japan) and the significance of spectra in HasApf/ImHApf were studied in detail. As shown in Figure 2, dried HasApf, immobilized HasApf (i.e., ImHApf), and Toyonite 200 were monitored via FTIR spectroscopy. Although the FTIR spectra of immobilized HasApf are similar to those of Toyonite 200, significant differences were detected in the range of 1750–1450 cm^−1^. This finding may be applicable to both the calibration of HasA in ImHApf and quality control of the ImHApf product.

#### 2.1.3. Construction of the HasA Calibration Curve in ImHApf

**Figure 3:**
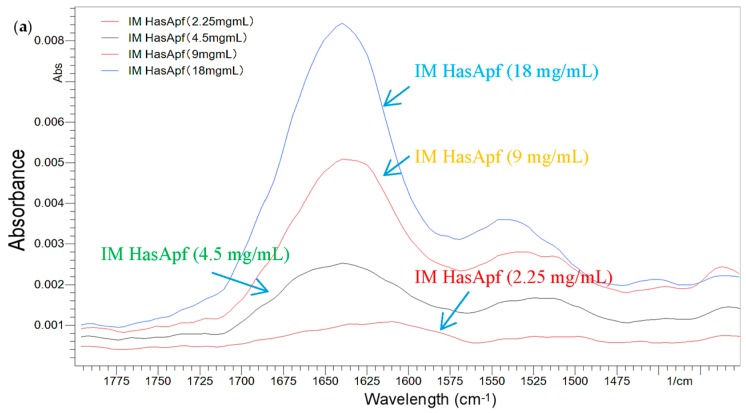

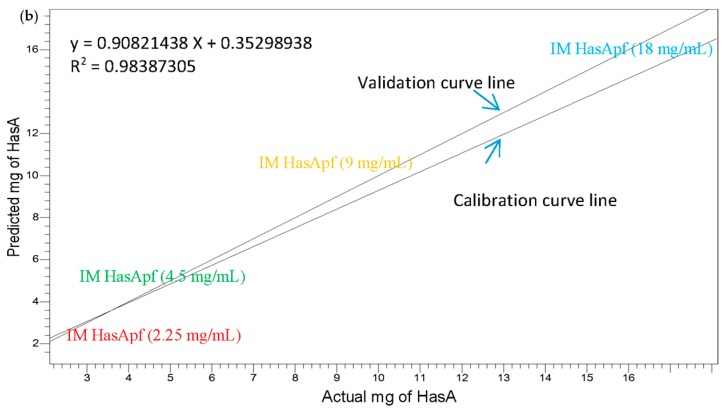
Construction of the HasA calibration curve in ImHApf. (**a**) FTIR spectra comparison among different HasA concentrations and (**b**) calibration curve line close to validation line, revealing HasA concentrations in ImHApf within the range of 1750–1450 cm^−1^: the two sets of data represent suitability for the immobilization to 16–18 mg HasA.

To further generate a calibration curve for the quality control of the product, ImHApf was prepared with different concentrations of HasA, and immobilization was performed using Toyonite 200 (1 g) per HasApf/potassium phosphate solution (2.25, 4.5, 9, and 18 mg/10 mL). As shown in Figure 3a, the concentration of HasApf in ImHApf was successfully monitored in the range of 1750–1450 cm^−1^. In addition, as shown in Figure 3b, the calibration curve was successfully constructed for the immobilization to 16–18 mg HasA [5], with predicted mg of HasA in the *y* axis and actual mg of HasA in the *x* axis. Thus, FTIR analysis is an ideal method for the quality control of the product because HasA leach can be experimentally evaluated after filtering the suspension (Figure 1). The advantages of immobilized ImHApf over the “free” ones in solution include stability, reusability, and convenience in continuous operation and are further examined below.

### 2.2. Catalytic Efficiency of ImHApf

#### 2.2.1. Continuous Reuse of ImHApf

**Figure 4:**
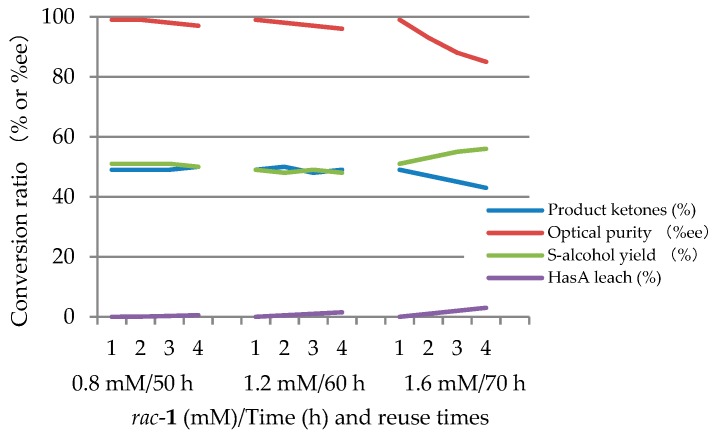
Number of reuses (1–4) of ImHApf (10 mg) under different substrate concentration/reaction time conditions (0.8 mM/50 h, 1.2 mM/60 h, and 1.6 mM/70 h), revealing that ImHApf can be continuously reused even if treated with an organic solvent.

ImHApf (10 mg) was applied with substrate concentrations/reaction times of 0.8 mM/50 h, 1.2 mM/60 h, and 1.6 mM/70 h in a 50 mM glycine–NaOH buffer (4.0 mL). After 50 h reactions, ketone-1 and (*S*)-**1** in mixture were extracted with hexane (4 mL), and additional *rac*-**1** was added to the mixture and allowed to incubate for an additional 50 h × 3. The results indicated that the activity was inversely proportional to the substrate concentration (0.8 mM/50 h > 1.2 mM/60 h > 1.6 mM/70 h), suggesting that the condition of 0.8 mM/50 h permitted reuse for three rounds of hexane extraction with stability and convenience in continuous operation. In addition, it was confirmed that, in the condition of ImHApf after multiple washing or reaction cycles using FTIR analysis, the HasA concentration was gradually reduced, suggesting that HasA was slightly leached from ImHApf (Figure 4). As shown in Figure 4, the isomer (*S*)-**1** (0.8 mM; 99% enantiomeric excess (ee), approximately 50% yield) was obtained from *rac*-**1** (1.6 mM) using ImHApf (10 mg), leaving the highly enantiopure *R*-(−)-**1** (>99% ee: approximately 50% chemical yield).

#### 2.2.2. Unit of Activity Regarding ImHApf

**Figure 5:**
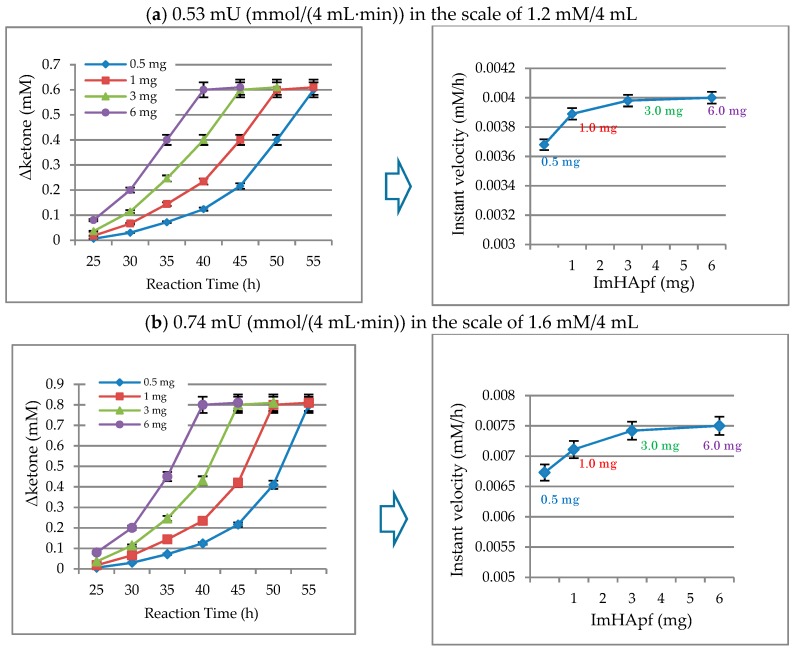
Comparison of the instant velocity (mM/h) per time (h) in the different concentrations of (**a**) 1.2 mM and (**b**) 1.6 mM between *rac*-**1** and ImHApf quantities (0.5, 1, 3, and 6 mg), i.e., the activity is 0.74 ± 0.03 mU/(mg·min), and the turnover number is approximately 2 × 10^6^. Error bars represent mean ± standard deviation (SD) (*n* = 4).

To investigate the activity regarding efficiency or turnover number, ImHApf was produced by suspending Toyonites (1.0 g) in a 50 mM buffer (pH 9) containing HasApf (18 mg/10 mL) for 50 h. The asymmetric oxidation activity of ImHApf (0.5, 1, 3, and 6 mg)/*rac*-**1** (1.2 mM or 1.6 mM) in a 50 mM glycine–NaOH buffer (pH 9.0; 4.0 mL) at 40 °C was examined under magnetic stirring (700 rpm, 40–60 h). Figure 5 (left side) shows the dependence of Δketone (mM) per time (h), which is the ketone concentration (mM) of the product on the reaction time (h) for each ImHApf quantity. In addition, Figure 5 (right side) shows the dependence of instant velocity (mM/h, referring to the maximum rate) on the reaction time (h) on each ImHApf quantity (mg). The data indicate the catalytic efficiency of HasA; thus, these results indicate that the activity of ImHApf is approximately 0.74 ± 0.03 mU/(mg·min), and the turnover number was determined to be approximately 2 × 10^6^. Thus, 0.5 mg of ImHApf containing approximately 0.0004 µM of enzyme catalyzes the formation of 0.8 mM ketone, placing the maximum observed number of turnovers per active site at 2 × 10^6^, as shown in Figure 5b. Therefore, HasApf immobilization utilizing Toyonite (0.74 mU) was beneficial to enzyme efficiency in a comparison to the previous results in a HasApf–complex eluted from pea protein (0.6 mU) [1].

### 2.3. Kinetic Resolution

#### 2.3.1. Substrate-Specific Reactivities to the *m*- and *p*-Substituted Groups

The substrate-specific reactivities of each enantiomer of *rac*-**1,** *rac*-**2,** and *m*- and *p*-substituted *rac*-1-phenyl ethanols (*rac*-**3ab**–**6ab**: 1.6 mM) on the oxidative activity of ImHApf (2 mg) were examined. After preincubation (1 h) of ImHApf with a 50 mM glycine–NaOH buffer (4.0 mL) at 40 °C on a magnetic stirrer at 700 rpm for 40–60 h, the solution reacted with racemic-**1** and -**2** and the other racemates (**3ab**–**6ab**). (*R*)-**1** and -**2** in racemic-**1** and -**2** oxidize highly enantioselectively by ImHApf; (*S*)-isomer was obtained with an enantiomer excess over 99% and a chemical yield of almost 50%. As shown in Table 1, it was confirmed that the (*R*)-isomers in *m*- and *p*-substituted *rac*-1-phenyl ethanols (*rac*-**3ab**–**6ab**: 1.6 mM) using ImHApf also oxidize enantioselectively to corresponding ketones; therefore, the other (*S*)-**3ab**–**6ab** could be obtained with high enantioselectivities. These results newly showed that the substrate-specific reactivity using ImHApf could be achieved not only in naphthyl groups (*rac*-**1** and -**2**) but also through the reaction of the racemate with *m*- and *p*-substituted aryl methyl carbinol (*rac*-**3ab**–**6ab**).

#### 2.3.2. ImHApf-Cyclic Deracemization Using NaBH_4_ (50 mg × 3)

After ImHApf (100 mg) was preincubated in a 50 mM glycine–NaOH buffer (300 mL) on a rotary shaker (55 rpm) at 40 °C for 1 h, the substrate (*rac*-**1** or -**2**: 45 mg) was mixed with the reaction solution and was further incubated for 40 h; then, an additional substrate (55 mg) was added and it was incubated for an additional 20 h. First results are reported in Table 2 in the substrate specificity of ImHApf, that is, only the (*R*)-isomer can be stereoselectively oxidized to the corresponding ketone, yielding a 50% chemical yield of (*S*)-isomer at >99% ee. Then, ImHApf-deracemization using NaBH_4_ (50 mg × 3) was performed in the one-pot incubations conducted in 10 h intervals in Table 2 (second, third and fourth). Table 2 shows that ImHApf (100 mg) can only oxidize the (*R*)-isomer to the correspondent ketone (50% chemical yield), which can be deracemized by NaBH_4_, thereby enabling obtainment in a single enantiomer ((*S*)-**1** or -**2**: >99% ee) and >90% ketone yield (<4%).

## 3. Discussion

ImHApf deracemization using NaBH_4_ was successful, and it is suggested herein that the HasA immobilization into porous ceramic particles may help to employ a deracemization process in conjunction with a chemical reducing agent for the production of biochemicals such as pharmaceuticals, agricultural chemicals, and their precursors [6]. The explorations of efficiency and turnover number are also needed for the application of ImHApf to organic synthesis [26]. In enzyme kinetics, it is interesting to clarify the maximum number of substrate molecules that can be converted into product per catalytic site for a given concentration of enzyme per unit time [27], although K_m_/V_max_ value is not applicable because of the lower substrate concentrations (<2 mM) and longer reaction times. The results in the present work newly indicate (1) the construction of a FTIR calibration curve using different concentrations of HasA in ImHApf; (2) an evaluation of the turnover number (approximately 2 × 10^6^) and activity [0.74 ± 0.03 mU/(mg·min)]; and (3) clarification of the cyclic deracemization process using ImHApf and NaBH_4_.

Therefore, the novelty of ImHApf deracemization featuring continuous reusability has recently been proposed for heterogeneous enzyme catalysis [28], in which a redox cofactor (i.e., heme) is immobilized to perform asymmetric oxidation with the advantages of stability, reusability, convenience in continuous operation, and volumetric productivity. In the future, the ability of HasA in ImHApf to enantioselectively oxidize (synthesizing optically active *sec*-alcohols) is expected to become an important tool for building an environmentally friendly system that promotes industrial sustainability.

## 4. Materials and Methods

### 4.1. Catalyst Preparation

#### 4.1.1. ImHApf Preparation with HasApf Protein Expression/Purification

HasApf was expressed in *E. coli* BL21(DE3) cells transformed with plasmid pET28a encoding bacterial genomic DNA (*P. fluorescens*) as previously reported [21]. For the preparation of immobilized HasA/ImHApf (approximately 0.0016 μM/mg), Toyonites (i.e., 200, 200A, 200P, or 200M: Toyo Denka Kogyo Co., Ltd., Kochi, Japan) (1.0 g) were suspended in a HasApf/potassium phosphate solution (18 mg/10 mL) for 50 h at room temperature, utilizing a Recipro shaker NR-10 (Taitec Co., Ltd., Saitama, Japan) with the speed set to 111 min^−1^. ImHApf was recovered by filtration, dried under vacuum [−50 °C/10 Pa (1 h) → 5 °C/min → +50 °C/10 Pa (3 h)] utilizing an RLE-II 203 instrument (Nissei Limited Co., Ltd., Tokyo, Japan), and stored in a desiccator. The filtrate was assessed using FTIR analysis for HasA leach (%), indicating the degree of HasA (%) that remains after filtrating the suspension.

#### 4.1.2. General Scale of the Reactions Using HasApf and ImHApf

The reaction mixture of ImHApf (0.5 mg–6.0 mg)/*rac*-**1**, -**2,** or -**3ab**–**6ab** (0.8 mg–1.6 mM) containing a 50 mM glycine–NaOH buffer (4.0 mL) in an 18 mm × 15 mL test tube was reacted at 40 °C under magnetic stirring at 700 rpm, i.e., the substrate solution (10 μL, 0.8 mM) that was prepared using *rac*-**1**, -**2**, or *rac*-**3ab**–**6ab** (1000 mg) in 2-propanol as a cosolvent (2.5 mL, 40,000 ppm) was dissolved, and the reactions were performed using HasA/ImHApf, approximately 0.0016 μM/mg/substrate (*rac*-**1**, -**2,** or -**3ab**–**6ab** (1.6 mM:0.8 mg)), in a 50 mM glycine-NaOH buffer (4.0 mL) for 50 h at suitable reaction conditions (namely, with a turnover number of approximately 2 × 10^6^). The reaction mixture containing a 50 mM glycine–NaOH buffer (300 mL) in an Erlenmeyer flask (500 mL) was reacted at 40 °C under magnetic stirring at 500 rpm by mixing the substrate solution (0.25 mL, 40,000 ppm) and ImHApf (100 mg).

#### 4.1.3. Enantiomeric Excess of Isolated Sec-Alcohols 

The calculations of enantiomeric excess, isolated from the reaction of *rac*-**1**, *rac*-**2**, or *rac*-*m*- and *p*-substituted *rac*-1-phenyl ethanols (*rac*-**3ab**–**6ab**: 0.8 mM or 1.2 mM) were performed for *rac*-**1** using a Daicel Chiralcel OB-H column (Tokyo, Japan), for *rac*-**2** using a Daicel Chiralpak AS-H column connected to a high performance liquid chromatography (HPLC) LC-10A system (Shimadzu Co., Ltd., Kyoto, Japan), and for *rac*-**3ab**–**6ab** using a gas chromatography with a Chrompack Chirasil-Dex CB capillary column (25 m 0.25 mm TC-5HT-fused silica-coated Agilent J & W CP Chirasil-Dex CB: Tokyo, Japan). The analytical conditions of *rac*-**1**, -**2**, or *rac*-**3ab**–**6ab**, including mobile phase, flow rate, and temperature, have been previously reported in detail [9,21].

#### 4.1.4. FTIR Analyses Using ImHApf

A portable type of ATR instrument was applied for FTIR analysis (A2 Technologies, ML version). After ImHApf samples were placed on the diamond crystal of ATR, FTIR spectra could be obtained via a pressure device (a single bounce reflection method), and the calibration curve of HasApf in ImHApf could then be constructed in the range of 1750–1450 cm^−1^ using Panorama software [15].

#### 4.1.5. Specific Activity and Turnover Efficiency on ImHApf

The activity of ImHApf was calculated using different concentrations of HasApf immobilized with Toyonite 200 (1 g) for each HasApf/potassium phosphate solution (18 mg/10 mL). The different volumes of ImHApf (0.5, 1, 3, and 6 mg) were reacted with *rac*-**1** (1.6 mM) in a 50 mM glycine–NaOH buffer (4.0 mL; pH 9.0) via magnetic stirring (700 rpm) for 0.8 mM/50 h, 1.2 mM/60 h, or 1.6 mM/70 h. This value indicates the quantity of enzyme (mg) that is capable of oxidizing 1 µmol of *rac*-**1** per minute. The formulae of instant velocity (mM/h), activity [mmol/(4 mL·min)], or specific activity {unit [mmol/(4 mL·min)]} have been previously reported [1].

## 5. Conclusions

ImHApf has continuous reusability in the asymmetric oxidation activity even if treated with an organic solvent, and its catalytic efficiencies are evaluated using the turnover number (approximately 2 × 10^6^) and activity [0.74 ± 0.03 mU/(mg·min)], suggesting that iron in ImHApf is oxidized by dissolved oxygen, leading to the promotion of oxoferryl (Fe(IV)=O) species. The kinetic resolution of (*S*)-(+)-**1** or *-***2** or *-***3ab**–**6ab** (>99% ee, approximately 50% chemical yield) from *rac-***1** or *-***2** or *-***3ab**–**6ab** catalyzes via the selective oxidation of (*R*)-(−)-**1** or *-***2** or *-***3ab**–**6ab** to the corresponding ketone. The ketone produced from (*R*)-(−)-**1** or *-***2** (100 mg) was deracemized by NaBH_4_ (50 mg × 3) using ImHApf (100 mg) and obtained as a pure enantiomer (>99% ee, approximately 90% chemical yield) in the absence of an added cofactor, e.g., NAD(P), in the aqueous medium at 40 °C. Therefore, HasAs with an immobilized porous ceramic particle are expected to become important asymmetric oxidation catalysts in organic synthesis, building environmentally friendly systems that promote industrial sustainability.

## Figures and Tables

**Table 1:** Asymmetric resolution of *rac*-ArCH(OH)Me (Ar = 2-Np and 6-MeO-2-Np, 3-Ar, and 4-Ar) with HasApf/ImHApf (2 mg, 0.0016 μM) under a substrate concentration (1.6 mM).

Substrate			Reaction Time (h)	Origin	Product		
Racemic-ArCH(OH)R	Ar	R			Compound	OP (% ee) ^a^	CY (%) ^b^
**1**	6-MeO-2-Np	Me	50	ImHApf ^c^	(*S*)-**1**	>99	50
**2**	2-Np	Me	55	ImHApf	(*S*)-**2**	>99	50
**3a**	3-Cl-Ar	Me	50	ImHApf	(*S*)-**3a**	>99	50
**4a**	3-Br-Ar	Me	55	ImHApf	(*S*)-**4a**	>99	49
**5a**	3-Me-Ar	Me	60	ImHApf	(*S*)-**5a**	>99	50
**6a**	3-MeO-Ar	Me	50	ImHApf	(*S*)-**6a**	>99	50
**3b**	4-Cl-Ar	Me	50	ImHApf	(*S*)-**3b**	>99	50
**4b**	4-Br-Ar	Me	55	ImHApf	(*S*)-**4b**	>99	50
**5b**	4-Me-Ar	Me	60	ImHApf	(*S*)-**5b**	>99	49
**6b**	4-MeO-Ar	Me	50	ImHApf	(*S*)-**6b**	>99	50

^a^ Optical purity (OP) determined by gas chromatograph. ee: enantiomer excess; ^b^ Chemical yield (CY); ^c^ Immobilized HasA from *Pseudomonas fluorescens*.

**Table 2:** Application of ImHApf to cyclic deracemization using NaBH_4_ (50 mg × 3).

	Deracemization: ImHApf (100 mg)/*rac*-1 (100 mg)/50 mM glycine–NaOH Buffer (300 mL)							
	NaBH_4_	Time (h)	% Ketone	% Alcohol ^a^	% *R*-isomer	% *S*-isomer	Compound	% ee
**1st**	0	60	50	50	1.6	98.4	(*S*)-1 ^b^	96.8
					1.2	98.8	(*S*)-2 ^c^	97.6
**2nd**	50 mg	80	32	68	1.1	98.9	(*S*)-1	97.8
					0.9	99.1	(*S*)-2	98.2
**3rd**	50 mg	90	17	83	0.7	99.3	(*S*)-1	98.6
					0.5	99.5	(*S*)-2	99.0
**4th**	50 mg	100	4	96	0.4	99.6	(*S*)-1	99.2
					0.2	99.8	(*S*)-2	99.6

ImHApf-deracemization can be achieved by NaBH_4_ addition. ^a^ Chemical yield (%); ^b^ 6-MeO-2-Np; ^c^ 2-Np.
